# Expression of glycerol-3-phosphate acyltransferase increases non-polar lipid accumulation in *Nannochloropsis oceanica*

**DOI:** 10.1186/s12934-022-01987-y

**Published:** 2023-01-16

**Authors:** Christian Südfeld, Aamna Kiyani, Katrin Wefelmeier, René H. Wijffels, Maria J. Barbosa, Sarah D’Adamo

**Affiliations:** 1 Wageningen University, Bioprocess Engineering, PO Box 16, 6700 AA Wageningen, Netherlands; 2 Department of Microbiology, Quaid-I-Azam University, Islamabad, 45320 Pakistan; 3 Faculty of Biosciences and Aquaculture, Nord University, N-8049 Bodø, Norway

## Abstract

Microalgae are considered a suitable production platform for high-value lipids and oleochemicals. Several species including *Nannochloropsis oceanica* produce large amounts of essential $\omega$-3 polyunsaturated fatty acids (PUFAs) which are integral components of food and feed and have been associated with health-promoting effects. *N. oceanica* can further accumulate high contents of non-polar lipids with chemical properties that render them a potential replacement for plant oils such as palm oil. However, biomass and lipid productivities obtained with microalgae need to be improved to reach commercial feasibility. Genetic engineering can improve biomass and lipid productivities, for instance by increasing carbon flux to lipids. Here, we report the overexpression of glycerol-3-phosphate acyltransferase (GPAT) in *N. oceanica* during favorable growth conditions as a strategy to increase non-polar lipid content. Transformants overproducing either an endogenous (NoGPAT) or a heterologous (*Acutodesmus obliquus* GPAT) GPAT enzyme targeted to the endoplasmic reticulum had up to 42% and 51% increased non-polar lipid contents, respectively, compared to the wild type. Biomass productivities of transformant strains were not substantially impaired, resulting in lipid productivities that were increased by up to 37% and 42% for NoGPAT and AoGPAT transformants, respectively. When exposed to nutrient stress, transformants and wild type had similar lipid contents, suggesting that GPAT enzyme exerts strong flux control on lipid synthesis in *N. oceanica* under favorable growth conditions. NoGPAT transformants further accumulated PUFAs in non-polar lipids, reaching a total of 6.8% PUFAs per biomass, an increase of 24% relative to the wild type. Overall, our results indicate that GPAT is an interesting target for engineering of lipid metabolism in microalgae, in order to improve non-polar lipid and PUFAs accumulation in microalgae.

**Supplementary Information:**

The online version contains supplementary material available at 10.1186/s12934-022-01987-y.

## Introduction

Photosynthesis is the process of capturing and using sunlight energy to assimilate CO_2_ into biomass. The ability of microalgae to grow photoautotrophically allows microalgal biomass production independent of energy-rich carbon-sources, which is a substantial advantage over production processes that use heterotrophic organisms. Microalgae can reach higher biomass productivities than traditional agricultural crops [1], and their production does not compete with food supply because they can be cultivated on non-arable land. Moreover, the ability of marine microalgal species to grow in salt-water uncouples photoautotrophic biomass production from fresh water supply, which will help to mitigate fresh water shortages in the future [2]. Oleaginous microalgal species can accumulate lipids to up to 60% on a dry cell weight (DCW) basis, and some species produce substantial amounts of high-value $\omega$-3 polyunsaturated fatty acids (PUFAs) that are relevant for application in food and feed [3, 4]. Therefore, microalgal oil has the potential to supplement agricultural production of food and feed, by replacing less sustainable oil sources. Moreover, microalgal oils are readily converted into biodiesel, and microalgal biodiesel has been hailed as a potential replacement for fossil fuels [1, 5].

The marine stramenopile microalga *Nannochloropsis oceanica* is a promising candidate for both, production of high-value lipids, and bulk commodities [3]. Upon nitrogen (N) deprivation, lipid contents of this species can reach up to 60% DCW ${}^{-1}$. However, productivities remain too low to allow for economically feasible lipid production [6, 7], partly because N deprivation reduces biomass production rates. Lipid productivities can be improved by genetic engineering, for instance, by uncoupling lipid accumulation from stress exposure [8–10]. To achieve this, a thorough understanding of the microalga’s lipid metabolism and its regulation are of pivotal importance.

The presence of the chloroplast in plants and microalgae has decisively influenced the compartmentalization of metabolic pathways, including lipid metabolism. Lipid metabolism in microalgae was recently reviewed in detail [11]. Unlike other eukaryotic organisms, which synthesize lipid building blocks, i.e. fatty acids (FAs), in the cytosol, photosynthetic organisms synthesize FAs in the chloroplast stroma [12]. In oleaginous organisms such as *Nannochloropsis*, FAs are stored in triacylglycerols (TAGs), which are the main energy storage compound, and present in cytosolic lipid droplets [13]. TAG synthesis in *Nannochloropsis* likely happens at the endoplasmic reticulum (ER), consistent with upregulation of ER-localized Kennedy pathway enzymes, and downregulation of their plastidic counterparts during nitrogen (N) starvation conditions [14, 15]. Incorporation of FAs into glycerolipids occurs via the Kennedy pathway, which begins with acylation of glycerol-3-phosphate by action of glycerol-3-phosphate acyltransferase (GPAT), using acyl-CoA as an acyl donor to produce lysophosphatidic acid (LPA). LPA is subsequently used as substrate of lysophosphatidic acid acyltransferase (LPAAT) together with a second acyl-CoA, producing phosphatidic acid. PA can either be used for synthesis of anionic phosphoglycerides such as phosphatidylinositol, or it can be dephosphorylated by action of phosphatidic acid phosphatase (PAP), producing diacylglycerol (DAG). DAG is a crucial branching point of the Kennedy pathway, as it serves as substrate for the synthesis of glycosylglycerides such as MGDG and DGDG, zwitterionic phosphoglycerides such as PC and PE, and the non-polar lipid (NL) TAG.

The final step of TAG assembly can occur through the acyl-CoA-dependent or independent pathway. The former involves diacylglycerol acyltransferase (DGAT) enzyme, which acylates DAG at the sn-3 position using acyl-CoA as acyl donor [16, 17]. The acyl-CoA-independent pathway utilizes phospholipids as an acyl donor, and utilizes phospholipid:diacylglycerol acyltransferase (PDAT). PDAT can transfer an acyl-moiety from the sn-2 position of e.g. PC to the sn-3 position of DAG, forming TAG and lyso-PC. Whereas PDAT is implicated in TAG synthesis mainly during favorable growth conditions, the bulk of TAG formation under N-depletion is catalyzed by DGAT in *Chlamydomonas* [18]. The *Nannochloropsis* genome contains 11 DGAT2 and two DGAT1 genes [19], four of which are predicted as ER-localized isoforms [14]. This substantial enrichment for DGAT genes compared to other organisms, was considered to be a potential explanation for the oleaginousness of this genus [19, 20]. Consequently, several studies have been conducted over the last few years, to evaluate the role of DGAT in lipid metabolism of *Nannochloropsis* by genetic engineering [21–25]. This research has shown that DGAT overexpression can lead to substantially increased TAG contents of transgenic strains, consistent with the central role of the Kennedy pathway in glycerolipid metabolism. Despite this, the role of GPAT in lipid metabolism of *Nannochloropsis*, has so far been neglected. As GPAT catalyzes the first committed and potentially rate-determining step of the Kennedy pathway [26], this enzyme may be a suitable target for genetically engineering *Nannochloropsis* strains with improved lipid content and productivity.

The aim of this study was to investigate the effect of GPAT overexpression on lipid accumulation in *N. oceanica*. For such purpose, we engineered *N. oceanica* to overexpress either the endogenous ER-localized GPAT isoform or a heterologous GPAT from the oleaginous microalga *Acutodesmus obliquus*. Physiological and biochemical characterization of transformant strains showed that GPAT is an important control point for lipid biosynthesis in *N. oceanica*.

## Results

### Design of GPAT expression constructs

**Fig. 1:**
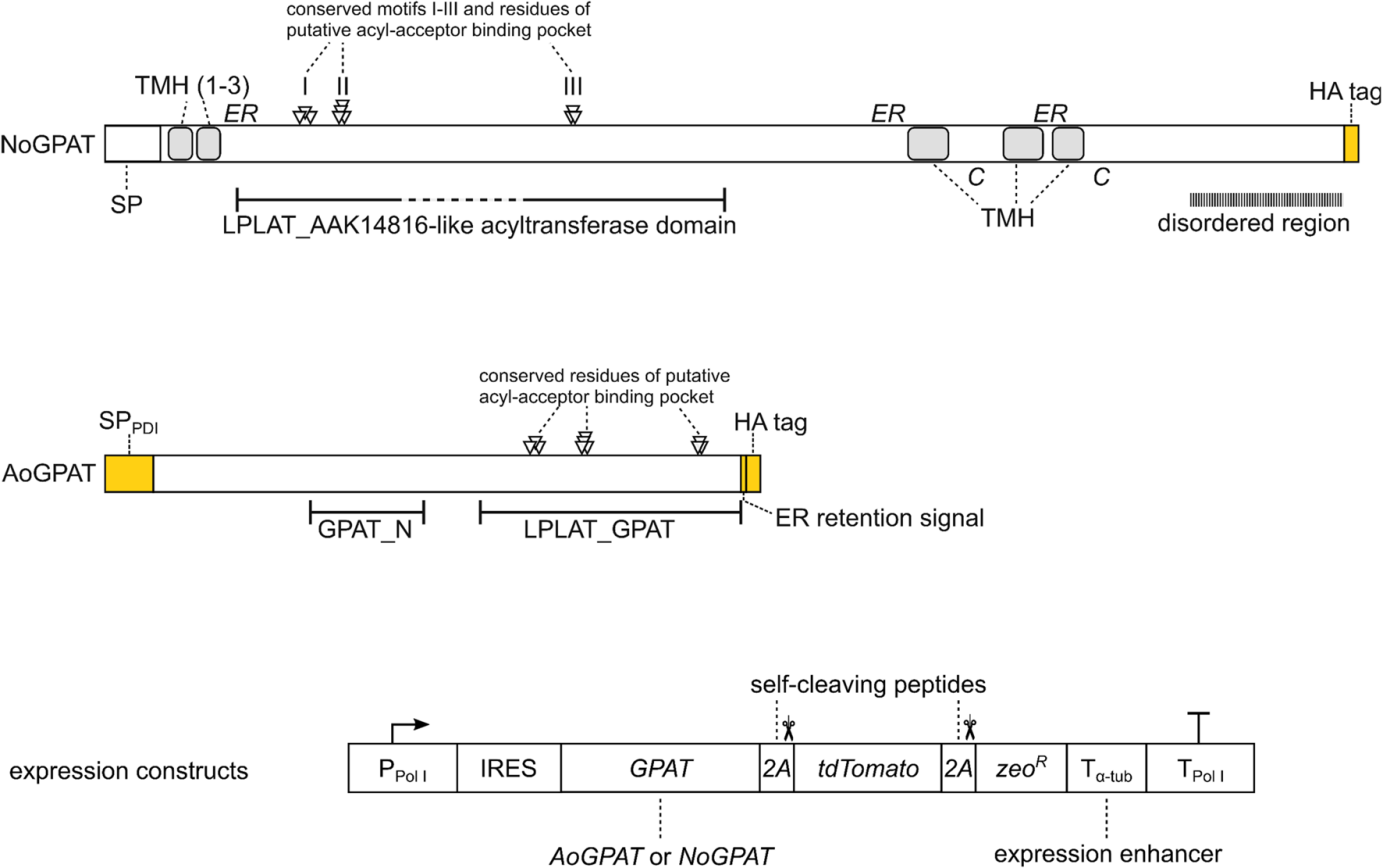
Design of GPAT-expression constructs for *N. oceanica.* Schematic of the ER-localized *N. oceanica* GPAT isoform NO03G04130 (NoGPAT) and the *A. obliquus* chloroplastic GPAT (AoGPAT) are shown together with a simplified schematic of the expression construct (not drawn to scale). Putative orientations of the different NoGPAT regions are indicated by *ER* or *C* for ER-lumenal and cytoplasmic orientation, respectively. Both genes were equipped with a C-terminal coding sequence for an HA tag, to allow immunodetection of proteins. Artificially added elements are colored yellow. *GPAT* genes were inserted into an expression construct based on a recently developed expression system [35], containing a Pol I promoter and terminator, an internal ribosome entry site (IRES) and an expression enhancer (T ${}_{\mathrm{\alpha }-\mathrm{tub}}$). A fluorescent reporter (*tdTomato*) and antibiotic resistance gene (*zeo* ${}^{R}$) were added for expression quantification and selection, respectively. The three genes were separated by coding sequences for self-cleaving 2A peptides to allow expression as a single cistron

The *N. oceanica* IMET1 genome contains two genes that are predicted to encode GPAT proteins, *NO03G04130* and *NO12G00610*. We analyzed both encoded proteins for similarities to known GPAT enzymes using BLASTp and we analyzed their domain architecture by querying the conserved domain database [27] and by using in silico protein analysis tools. NO12G00610 is a homologue of plastidic GPAT proteins of higher plants (38.02% identity on 56% coverage with Squash plastidic GPAT, E value = 7 $\times {10}^{-52}$; 36.75% identity on 63% coverage with soybean plastidic GPAT, E value = 3 $\times {10}^{-56}$) and the protein is predicted to localize to the chloroplast in *N. oceanica*. NO03G04130 is a homologue of ER-localized GPAT enzymes of other stramenopile organisms and fungi, including SCT1 proteins from *Saccharomyces cerevisiae* (34.77% identity on 76% coverage, E value = 9 $\times {10}^{-94}$) and *Schizosaccharomyces pombe* (33.21% identity on 76% coverage, E value = 4 $\times {10}^{-81}$), as well as GPT2 protein from *S. cerevisiae* (29.71% identity on 77% coverage, E value = 6 $\times {10}^{-69}$). NO03G04130 was previously shown to localize to the ER in *N. oceanica* [15]. Analogous to its yeast counterparts, NO03G04130 contains a 1-acyl-sn-glycerol-3-phosphate acyltransferase domain (Fig. 1) with high sequence similarity to LPLAT_AAK14816-like domains (domain subfamily cd07992). This kind of domain is involved in transfer of acyl groups from acyl-CoA or acyl-ACP to glycerol 3-phosphate, dihydroxyacetone phosphate or lyso-phosphatidic acid and it contains 4 conserved sequence motifs I, II, III, and IV [28, 29]. Based on the crystal structure of a chloroplastic GPAT from *Cucurbita moschata* (acyltransferase family cd07985), motifs I, II and III are predicted to form a surface pocket that binds G3P or other substrates [30, 31]. All ten conserved residues of motifs I-III are present in NO03G04130, whereas motif IV is absent, which is frequently the case for proteins containing domain cd07992. NO03G04130 is predicted to contain between four and six transmembrane helices, which likely orient the conserved acyltransferase domain to the ER lumen [28].

Li et al. reported that the majority of ER-localized Kennedy pathway enzymes including *NO03G04130* are upregulated in *N. oceanica* during N stress, whereas most of the plastidic isoforms (including *NO12G00610*) are downregulated [14]. NO03G04130 is thus far the only identified extraplastidic GPAT of *N. oceanica* and a previous attempt to generate *NO03G04130* knockout transformants failed, indicating that this enzyme is non-redundant and essential for cell survival [15]. We, therefore, decided to focus our investigations on the ER isoform *NO03G04130*, hereafter referred to as *NoGPAT*. NoGPAT contains an LPLAT_AAK14816 acyltransferase domain that is frequently found in eukaryotic ER-localized GPATs. All ten conserved residues of this domain type are present in the three conserved motifs I, II and II (Fig. 1). A signal peptide (SP) directs the enzyme to the ER, and four to six transmembrane helices (TMH) anchor the enzyme in the ER membrane, likely orienting the acyltransferase domain to the ER lumen. The C-terminal domain contains a disordered region, which likely faces the cytoplasm.

ER-localized GPAT enzymes are reported to be subjected to transcriptional, post-transcriptional and post-translational regulation [26, 29, 32]. To overcome the possibility of negative endogenous regulation, we decided to test heterologous expression of a *GPAT* gene (*AoGPAT*) from the oleaginous green microalga *A. obliquus*. This enzyme was recently heterologously expressed in the microalga *Neochloris oleoabundans*, which resulted in elevated TAG and PUFA contents [33]. AoGPAT is a homologue of the chloroplastic *C. reinhardtii* GPAT CrGPATcl (71.43% identity on 68% coverage, E value = 3 $\times {10}^{-123}$) and it contains an acyltransferase domain in its C-terminal region and an N-terminal domain frequently found in chloroplastic GPAT enzymes of plants (Fig. 1). To facilitate localization of *AoGPAT* to the ER, the gene was modified to encode the signal peptide of the endogenous ER-localized protein disulphide isomerase (SP ${}_{\mathrm{PDI}}$, Fig. 1), and a C-terminal ER retention signal [34].

We integrated the intron-free coding sequences of *NoGPAT* and the modified *AoGPAT* into an expression vector that utilizes a recently developed gene expression system [35]. This gene expression system uses an RNA polymerase I (Pol I) promoter, an internal ribosome entry site (I) and a 3’-expression enhancer sequence (T ${}_{\alpha -tub}$) to facilitate strong transgene expression. The human influenza hemagglutinin (HA) tag coding sequence was added to the 3’-ends of *NoGPAT* and *AoGPAT*, and the genes were inserted into the expression vector between the internal ribosome entry site coding sequence and the fluorescent reporter gene *tdTomato*. Expression of separate GPAT and tdTomato proteins was safeguarded by inserting a self-cleaving 2A peptide coding sequence between the genes [36]. A second 2A peptide coding sequence further allowed expression of the zeocin antibiotic resistance gene *zeo* ${}^{R}$ from the same cistron. The Pol I promoter and terminator sequences acted as homology arms that directed the construct to the rDNA cistron of chromosome 3 (Additional file 1: Fig. S1a), which we recently identified as a genomic safe harbor, conferring high gene expression [35].

### Engineering *Nannochloropsis oceanica* strains overexpressing either *GPAT* gene

**Fig. 2:**
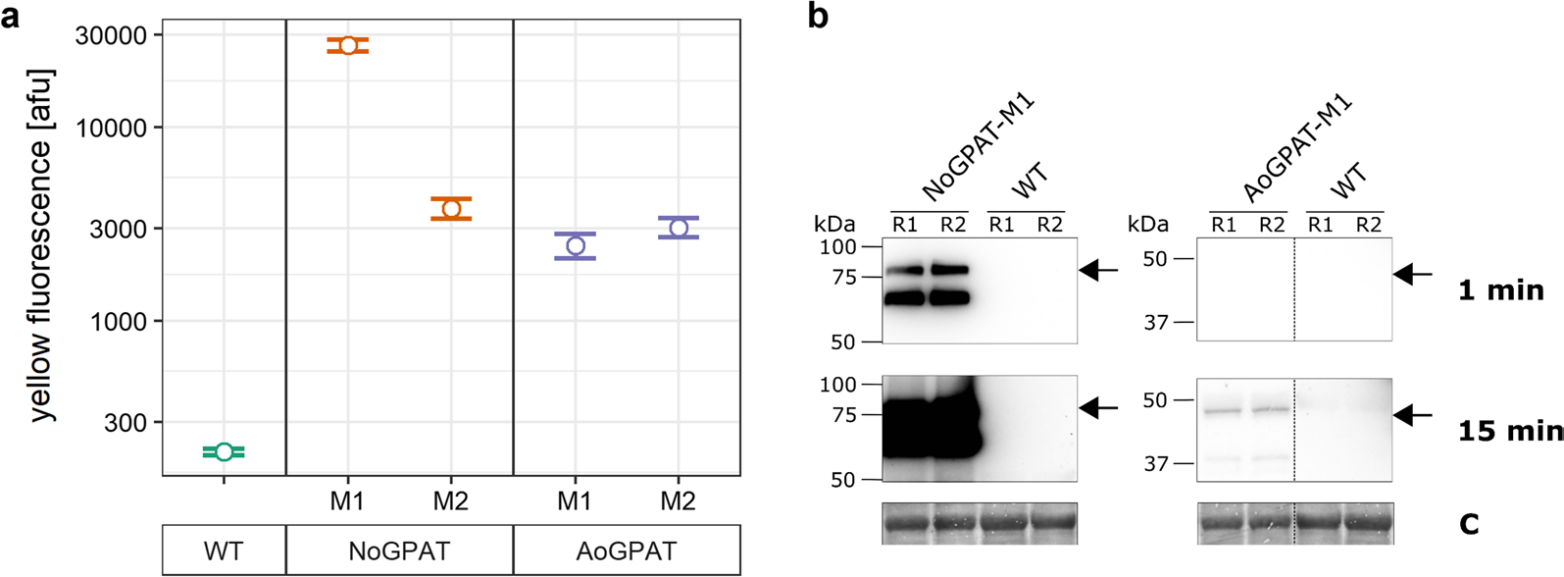
Analysis of gene expression in overexpression mutants. **a** Quantification of reporter fluorescence by flow cytometry. Yellow fluorescence levels, corresponding to tdTomato fluorescence emission, were increased in all mutant strains. Fluorescence levels of NoGPAT-M1 and M2 were higher than for AoGPAT mutants, which suggests stronger expression of the endogenous *GPAT* compared to the heterologous gene. TdTomato fluorescence of NoGPAT-M2 was substantially decreased compared to NoGPAT-M1, likely due to the partial *tdTomato* gene sequence deletion found for this mutant (Additional file 1: Fig. S1b). **b** Western blot analysis of *GPAT* gene expression for NoGPAT-M1 and AoGPAT-M1. Protein extract was separated by SDS-PAGE, and recombinant GPAT was detected by immunoblotting for two biological replicates (R1 and R2) of NoGPAT-M1 and AoGPAT-M1 with 1 min and 15 min exposure times during chemiluminescence detection, on a single membrane. In line with the results of flow cytometry analysis, chemiluminescence signals were substantially higher for NoGPAT-M1 compared to AoGPAT-M1, suggesting more efficient expression of the endogenous gene compared to the heterologous one. The RuBisCO large subunit band of the Coomassie-stained membrane (C) is shown as a loading control

*N. oceanica* was transformed with both constructs separately, and mutant colonies were grown on antibiotic containing agar plates. We then selected two colonies per construct that showed tdTomato fluorescence on agar plates, for further analysis. Genotyping PCR (Additional file 1: Fig. S1b) revealed that the construct had been integrated at the safe harbor site of chromosome 3 in all mutants. In line with this, we found increased reporter fluorescence levels compared to the wild type for all strains (Fig. 2a). Sequencing of PCR products further revealed a partial deletion in the *tdTomato* sequence of NoGPAT-M2. This deletion caused a removal of 242/476 amino acids of full-length tdTomato in NoGPAT-M2, which roughly corresponds to one monomer of the tandem dimer protein. Consequently, fluorescence of NoGPAT-M2 cells was substantially decreased compared to NoGPAT-M1 (Fig. 2a), but still higher than autofluorescence of the wild type. The rest of the cassette had remained intact, so this deletion should not affect NoGPAT expression or functionality.

Interestingly, AoGPAT mutants showed lower levels of tdTomato fluorescence than NoGPAT mutants. Similarly, AoGPAT protein from AoGPAT-M1 was detectable by immunoblotting only after substantially longer chemiluminescence exposure compared to NoGPAT protein from NoGPAT-M1 (Fig. 2b). These results suggest that expression of the *A. obliquus GPAT* gene may be reduced in *N. oceanica* compared to expression of the endogenous gene, which could be linked to sub-optimal codon usage of the heterologous gene, or to regulatory mechanisms that affect transcript processing, transcript translation, or protein stability. In this context, a recent study has shown that expression of a heterologous reporter gene was greatly enhanced in *N. oceanica*, when the 42 5’-terminal bases of the endogenous nitrate reductase coding sequence were added to the 5’-terminus of the heterologous gene, to facilitate formation of a fusion protein of the heterologous reporter with an endogenous leader sequence [37]. Expression of AoGPAT might be improved using a similar approach.

### Physiological characterization of GPAT-overexpressing strains

**Fig. 3:**
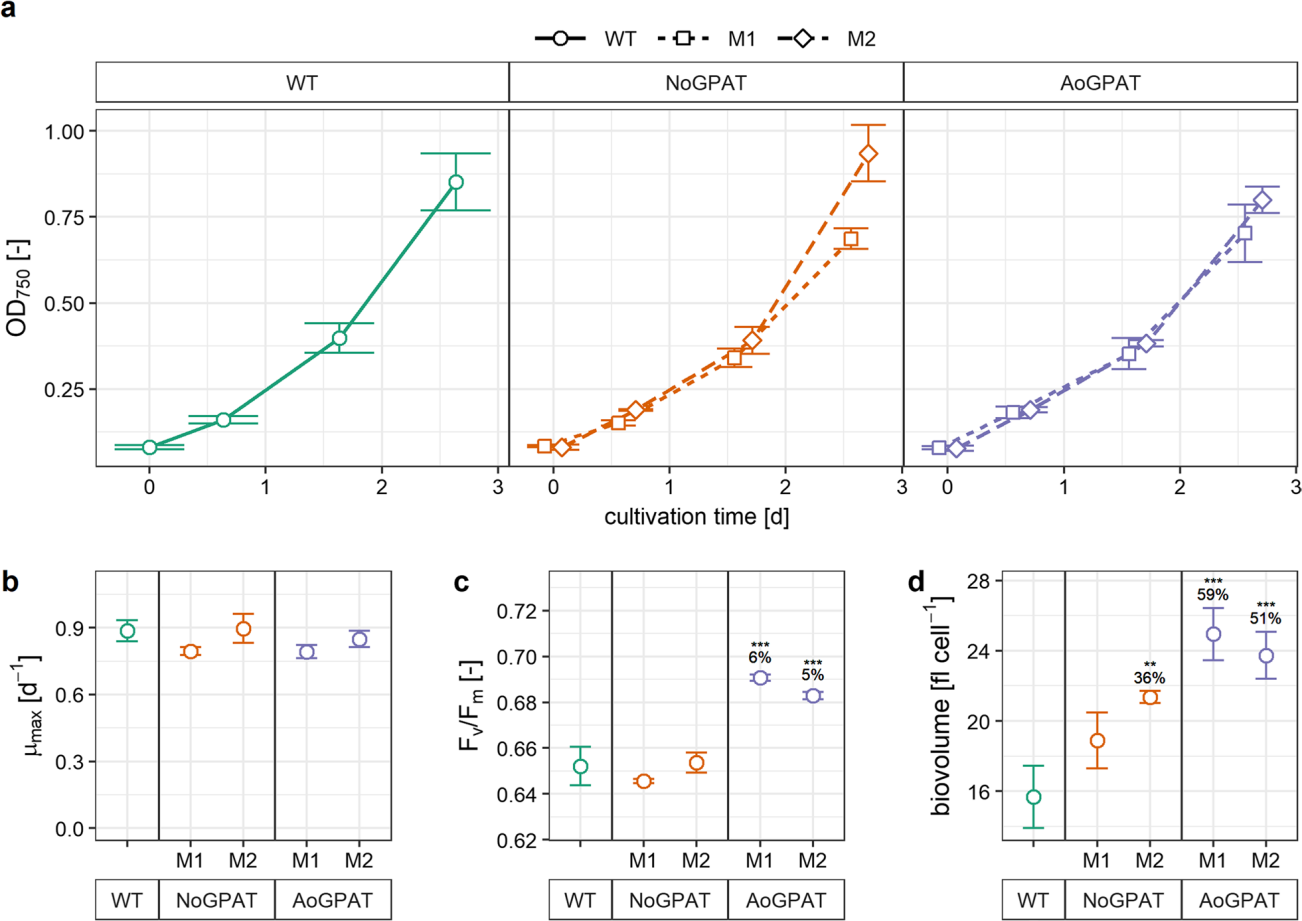
Physiological characterisation of mutant strains. **a** Growth curves of *N. oceanica* NoGPAT and AoGPAT mutants. Growth of mutant strains was comparable to each other and to the wild type. All strains grew exponentially for 3 days and reached comparable biomass densities. **b** Maximum specific growth rate of mutant strains, calculated using data shown in **a**. Despite a significant ANOVA, a post-hoc test revealed no significant difference between the wild type (WT) and any mutant. **c** Maximum quantum efficiency of photosystem II photochemistry of microalgal cultures, averaged over 3 days of exponential growth. **d** Average cellular biovolume of mutant strains after 3 days of exponential growth. **a**–**d** Data shown are the mean $\pm$ SD of N = 3 biological replicates (N = 5 for WT). **c**, **d** Relative differences compared to the WT are indicated above groups. Statistically significant differences between all groups were assessed using two-way ANOVA. In case of a significant ANOVA outcome, significant differences between means of individual groups and the wild type control were calculated using Tukey’s HSD test, and are indicated by asterisks. (*): $p<0.05$; (**): $p<0.01$; (***): $p<0.001$

We compared the growth of the NoGPAT and AoGPAT mutants (two mutants each) in a batch cultivation, using a parallel screening photobioreactor operated with diurnal light cycles and 600 $\mu \mathrm{mol}\hspace{0.25em}{\mathrm{m}}^{-2}\hspace{0.25em}{\mathrm{s}}^{-1}$ light intensity. Growth of these mutants was comparable to the wild type (WT), and growth was exponential over a 3 days cultivation period (Fig. 3a, N $\ge 3$). No mutant showed a significant difference in maximum specific growth rate compared to the wild type (Fig. 3b). Average cellular biovolume after 3 days of cultivation was 21–36% increased for NoGPAT and 51–59% increased for AoGPAT mutants (Fig. 3d), relative to the wild type. Moreover, AoGPAT-M1 and M2 showed 6 and 4% increased maximum quantum efficiency of photosystem II photochemistry (Fv/Fm), respectively (Fig. 3c). We further analysed growth upon exposure to nitrogen deprivation stress, and we found no significant differences between any of the strains and the wild type (Additional file 2: Fig. S2a). Higher Fv/Fm values of AoGPAT mutants were retained under nitrogen deprivation (Additional file 2: Fig. S2b).

### Analysis of lipid contents and fatty acid compositions

**Fig. 4:**
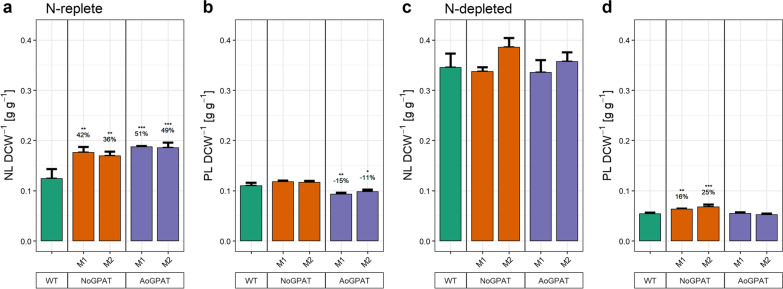
Lipid contents of mutant strains during exponential growth and after exposure to N-depletion stress. **a, b** Non-polar lipid (NL, a) and polar lipid (PL, b) contents of mutant strains during exponential growth phase. NoGPAT and AoGPAT mutants had markedly increased NL content, whereas PL contents were similar to the wild type (NoGPAT) or slightly decreased (AoGPAT). **c, d** NL and PL contents after exposure to 2 days of N-depletion stress. No mutant had significantly different NL content compared to the wild type. PL contents were slightly higher for NoGPAT-M1 and M2, compared to the wild type, but substantially decreased compared to N-replete cultivation. (a-d) Relative differences compared to the WT are indicated above groups. Data shown are the mean $\pm$ SD of N = 3 biological replicates (N = 5 for WT). Statistical significance was assessed by Tukey’s HSD test, in case of a significant ANOVA outcome. (*): $p<0.05$; (**): $p<0.01$; (***): $p<0.001$

Next, we quantified total non-polar lipid (NL) and total polar lipid (PL) content of mutant strains grown under N-replete and N-depleted conditions. No substantial differences were observed for NL content of N-depleted cultures (Fig. 4c), whereas PL content of NoGPAT-mutants was increased by 16–25% (Fig. 4d).

During replete conditions, NL content was increased by 36–42% for NoGPAT-M1 and M2, and by 49–51% for AoGPAT-M1 and M2 (Fig. 4a). Total PL contents were not significantly different between wild type and NoGPAT mutants (Fig. 4b), but decreased by 11–15% in AoGPAT-M1 and M2.

**Fig. 5:**
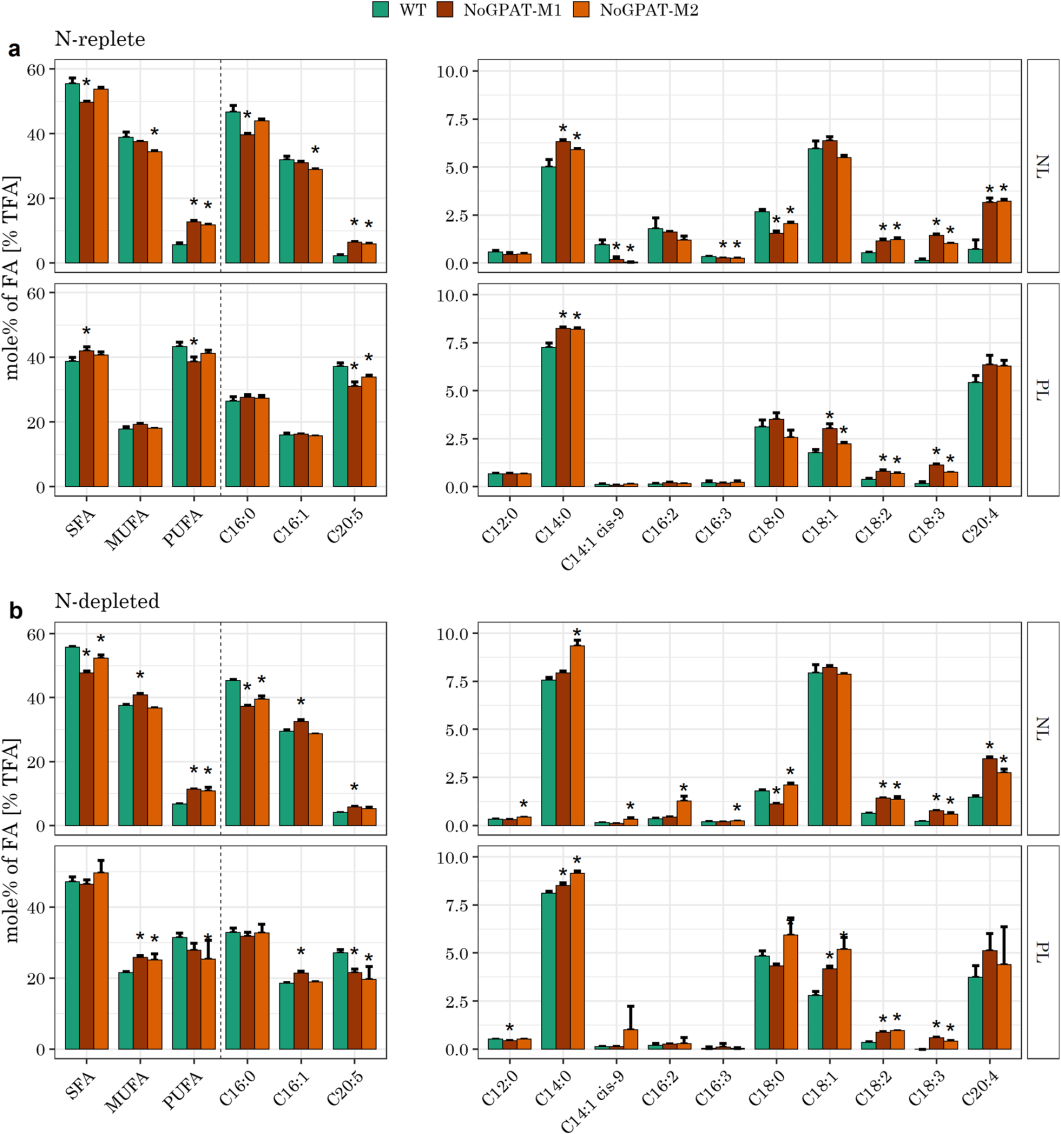
Fatty acid profiles of NLs and PLs for *N. oceanica* NoGPAT expression mutants. **a** FA compositions of NLs and PLs for exponentially growing cultures. FA contents are normalized to the total FA content per lipid class. NoGPAT-M1 and M2 showed a decrease in SFAs and an increase in PUFAs in the NLs. C18 PUFAs were further increased in PLs for these mutants, whereas C20:5 was depleted in PLs. **b** FA compositions for cultures after exposure to 2 days of N-depletion. NoGPAT-M1 and M2 showed an increased fraction of PUFAs and decreased fraction of SFAs, similarly to non-stressed conditions. Moreover, these mutants showed an enrichment of MUFAs in PLs, which was not seen for unstressed cultures. (a-b) Data shown are the mean $\pm$ SD of N = 3 biological replicates (N = 5 for WT). Statistical significance was assessed by Tukey’s HSD test. (*): $p<0.001$

We further analyzed the FA profile of both lipid classes. AoGPAT mutants did not show relevant changes in fatty acid composition under either condition (Additional file 3: Fig. S3). By contrast, NoGPAT, M1 and M2 showed increases in PUFAs in NL fractions (Additional file 4: Fig. S4e). Specifically, C18:2, C18:3 and C20:4 were significantly increased, compared to wild type, during both nitrogen-replete and deplete conditions. Under N-replete conditions, C20:5 was increased by 155 and 184% in the NL fraction for M1 and M2, respectively, but it was decreased in the PL fraction. Under N-replete conditions, both mutants also showed a significant decrease in C16:0 and C18:0 in NLs, whereas abundance of C14:0 was increased in both NLs and PLs (Fig. 5a).

Moreover, the fraction of C20:4 was increased by 340 and 349% in NLs for M1 and M2, respectively, and it was unchanged in PLs, in both N replete and deplete conditions. N-depleted NoGPAT mutants additionally showed an increase in C18:1 in PLs, which was not observed for N-replete conditions (Fig. 5).

These observations suggest that NoGPAT may have a substrate preference for long chain PUFAs or that its overexpression increases long chain PUFA synthesis in the ER.

Overall, NoGPAT mutants had increased lipid and PUFA productivities compared to the wild type, whereas AoGPAT mutants had an increased lipid productivity (Additional file 5: Table S1).

## Discussion

Despite the considerable research into LPAAT and DGAT enzymes [15, 21–25], the role of GPAT enzymes in lipid metabolism of *Nannochloropsis* is understudied. Here we have shown that GPAT overexpression can improve growth-associated NL accumulation in *N. oceanica*. NL content was increased in overexpression mutants only under replete conditions, and not after exposure to N-depletion. This suggests, that GPAT availability or regulation can exert strong flux control for NL synthesis during exponential growth, but not after exposure to nutrient stress. This is in accordance with the transcriptional upregulation of endogenous ER-localized GPAT in *N. oceanica* during N-depletion [14], which may increase GPAT availability or activity to levels that allow increased carbon flux towards TAGs. Our findings are in line with previous findings, showing increased TAG accumulation in GPAT-overexpressing transformants of other microalgal species [33, 38–42]. Our results show that growth-associated lipid accumulation in *N. oceanica* can be enhanced by expression of a single rate-determining enzyme. Accordingly, lipid production processes in continuous operation might become feasible by optimizing this microalga through metabolic engineering. Continuous operation circumvents the negative impacts of nutrient starvation on biomass and lipid productivities, and can therefore be desirable compared to 2-step processes [43, 44].

Interestingly, overexpression of the endogenous GPAT *NO03G04130* and heterologous expression of a GPAT gene from *A. obliquus* had similar effects on NL contents and productivities of transformants in our experiments (Additional file 1: Table S1), suggesting that heterologous enzymes might be able to functionally complement or substitute endogenous counterparts in *Nannochloropsis*. This opens up avenues for sophisticated synthetic biology strategies in this organism, in order to manufacture microalgal strains with FA and lipid metabolism geared towards production of “designer lipids”. The differences that were observed between NoGPAT and AoGPAT mutants in terms of FA profile, photosynthetic performance, and PL content during N-depletion, may be related to differences in enzyme characteristics such as membrane integration, substrate preference, and kinetic parameters, or to different expression levels.

It was previously shown that overexpression of endogenous GPAT genes increased PUFA contents in the diatom *Phaeodactylum tricornutum* [38, 40]. Similarly, overexpression of *NoGPAT* in this study shifted the FA composition especially of non-polar lipids to higher fractions of different long chain (LC) and very long chain PUFAs (VLC-PUFAs, Fig. 5). VLC-PUFAs such as C20:5 (EPA) are considered high-value compounds that are associated with health benefits in humans, and they are an essential component of aquaculture feed [4, 45, 46]. LC-PUFAs such as C18:3 further have potential for treatment and prevention of inflammatory disorders, cardiovascular disorders, cancer, and diabetes [47–49]. Therefore, the increased PUFA content of NoGPAT-M1 and M2 is intriguing (Additional file 5: Table S1). The substantially increased fraction of C18:2, C18:3, C20:4 and C20:5 in NLs of these mutants suggests that increased NoGPAT activity stimulates PUFA synthesis. In *Nannochloropsis*, an ER-localized pool of the glycerophospholipid phosphatidylcholine (PC) is likely the carrier for desaturation reactions of C18, whereas phosphatidylethanolamine (PE) and/or the betaine lipid diacylglyceryltrimethylhomoserine (DGTS) were suggested as carriers for C20 desaturation reactions [50, 51]. Current models for PUFA synthesis in microalgae suggest that desaturation of C18:1 ${}^{\Delta 9}$ bound to the sn-2 position of PC by action of $\Delta 12$-FAD leads to the formation of C18:2 ${}^{\Delta \mathrm{9,12}}$. C18:2 ${}^{\Delta \mathrm{9,12}}$ is the branching point for subsequent elongase and desaturase reactions involved in the formation of omega-6 or omega-3 fatty acids, which are then incorporated into DAG via the Kennedy pathway [11, 20, 50].

DAG is a branching point for de novo synthesis of glycerolipids like PC, PE, DGTS and for TAG. The central role of DAG illustrates the significance of GPAT in lipid and PUFA metabolism, as GPAT initiates the de novo synthesis of DAG. An increased level of DAG synthesis through action of GPAT might increase synthesis of other ER-located glycerolipids such as PC and PE, and thereby the availability of substrate for FAD enzymes. In this context, a previous study has shown that PUFA synthesis in *N. oceanica* is not limited by abundance of $\Delta 12$-FAD, which catalyzes the desaturation of PC-bound C18:1 to C18:2, exemplified by similar FA compositions of $\Delta 12$-FAD overexpression transformants and the wild type under N-replete conditions [52]. Therefore, further studies should focus on quantification of different lipid classes, including glycolipids, and on analysis of the glycerolipid-specific FA composition and their stereochemical distribution, to further elucidate the roles of different lipid pools for PUFA synthesis in NoGPAT-M1 and M2. The increased abundance of certain FAs in NoGPAT-M1 and M2 may further be due to a preference of NoGPAT for these species, which could be investigated by heterologous expression studies in yeast, or by in vitro assays, although in vitro enzyme specificity does not necessarily reflect in vivo conditions [53]. Notably, C18:2, C18:3 and C20:4 were increased in both, NLs and PLs of NoGPAT-M1 and M2, accounting for an overall increase of PUFAs in these mutants, compared to wild type (Fig. 5a, Additional file 4: Fig. S4). Similarly, a recent study by Poliner and colleagues [54] has shown that simultaneous overexpression of $\Delta 5$, $\Delta 9$ and $\Delta 12$-FAD in *N. oceanica* increased fractions of C18:2 and C20:4 in TLs by 125% and 73%, respectively.

It should further be noted that a growing body of evidence suggests a role of Kennedy pathway intermediates in intracellular signaling cascades [55, 56]. Consequently, NoGPAT overexpression may results in differential expression of other FA or lipid metabolism-related genes, similarly to what was recently reported for a GPAT and LPAAT overexpression transformant of the diatom *Phaeodactylum tricornutum* [57]. Transcriptomic analyses of NoGPAT-M1 and M2 may help to unravel the mechanism behind the altered PUFA synthesis in these transformants.

AoGPAT-M1 and M2 showed significantly increased photosynthetic efficiency compared to all other strains (Fig. 3c). This was accompanied by increases in NL contents (Fig. 4a) and average cell size (Fig. 3d), whereas PL contents were decreased (Fig. 4b). Future studies should investigate a possible connection between the decreased PL content and the increased photosynthetic efficiency and NL contents of AoGPAT mutants. These kind of studies may include quantification of PL classes and detailed characterization of photosynthetic parameters. In this context, a recent study has shown that a *Nannochloropsis gaditana* mutant that was likely impaired in synthesis of the main photosynthetic PLs MGDG and DGDG, displayed an increased proton motif force across the thylakoid membrane and an increased TL content under N-replete, but not N-depleted conditions [58].

## Conclusion

In this study we genetically engineered *N. oceanica* transformant strains to express ER-targeted GPAT enzymes. Transformants showed marked increases in NL contents and productivities under replete conditions, with little effect on growth. The endogenous GPAT *NO03G04130* and a heterologous GPAT gene from *A. obliquus* were both successfully expressed, and expression of both enzymes had similar effects on lipid contents of transgenic strains. Overexpression of *NO03G04130* further resulted in an increase in PUFA content, especially of C18 species and C20:4. Expression of the GPAT gene from *A. obliquus* instead resulted in an increase in photosynthetic performance and a concomitant decrease in PLs. Concluding, we have shown that ER-localized GPAT enzyme is an interesting target for genetic engineering of improved NL and PUFA production in *N. oceanica*, and potentially also in other oleaginous microalgae. These insights will help to transform *Nannochloropsis* into a viable production platform for lipids and value-added fatty acids. However, further studies are needed to elucidate the role of GPAT in lipid accumulation in *Nannochloropsis*, and to identify additional metabolic bottlenecks that limit carbon flux to lipids under favorable growth conditions.

## Materials and methods

### Microalgal strains and cultivation

The microalga used in this study was *N. oceanica* IMET1, which was a kind gift by prof. Jian Xu (Qingdao Institute for Bioenergy and Bioprocess Technology, Chinese Academy of Sciences). Microalgae were cultivated in artificial sea water (ASW, 419.23 mM NaCl, 22.53 mM Na ${}_{2}$ SO ${}_{4}$, 5.42 mM CaCl ${}_{2}$, 4.88 mM K ${}_{2}$ SO ${}_{4}$, 48.21 mM MgCl ${}_{2}$ and 20 mM HEPES, pH 8), supplemented with 2 ml ${\mathrm{l}}^{-1}$ of Nutribloom plus (Necton, Portugal), at 25 ${}^{\circ }\mathrm{C}$. For physiological and biochemical characterization, we used an Algem HT24 photobioreactor (Algenuity, UK) that was placed inside an HT Multitron Pro (Infors Benelux, Netherlands) orbital shaker unit with 0.2% CO ${}_{2}$-enriched air at 120 rpm shaking frequency. All experiments were carried out with a 16:8 h diurnal light cycle. Sampling was carried out 1.5 h before dusk.

For cultivation on solid medium, ASW was supplemented with 1% (w/v) of agar and mixed with 2 ml ${\mathrm{l}}^{-1}$ of nutribloom plus and 5 $\mu \mathrm{g}\hspace{0.25em}{\mathrm{ml}}^{-1}$ zeocin after autoclaving. Microalgal transformant plates were incubated at 25 ${}^{\circ }\mathrm{C}$ in ambient air using warm-white fluorescent light bulbs and an illumination intensity of 60–80 $\mu \mathrm{mol}\hspace{0.25em}{\mathrm{m}}^{-2}\hspace{0.25em}{\mathrm{s}}^{-1}$.

### Construction of transformation constructs

The two GPAT genes were cloned into a previously reported vector pCS-EC6 [35] using Gibson assembly technique (NEBuilder HiFi DNA Assembly Master Mix, NEB #E2621). Fragments were amplified using PCR primers shown in Additional file 5: Table S2. NoGPAT fragments were amplified from *N. oceanica* genomic DNA that was extracted as previously described [10]. AoGPAT was amplified from pUC-AcobLPAT [33]. Linear expression constructs were amplified from restriction-linearized plasmids pCS-EC6-NoGPAT and pCS-EC6-AoGPAT using primers oCSG13 and oCSG14. All PCRs for cloning purposes were carried out using Q5 DNA polymerase (NEB #M0492). Genotyping PCRs were carried out using Phire DNA polymerase (Thermo Fisher Scientific #F160).

### Transformation of *N. oceanica*

*N. oceanica* IMET1 was transformed using electroporation as previously reported [20]. Briefly, microalgal cells were cultivated at 150 $\mu \mathrm{mol}\hspace{0.25em}{\mathrm{m}}^{-2}\hspace{0.25em}{\mathrm{s}}^{-1}$ illumination for at least 3 days, and harvested during mid-exponential growth stage by centrifugation (2,500 × g, 5 min, 4 ${}^{\circ }\mathrm{C}$). Pellets were washed thrice with 375 mM sorbitol (4 ${}^{\circ }\mathrm{C}$) and resuspended at 2.5 × 10 ${}^{9}$ cells ml ${}^{-1}$. 200 ﻿$\mu$l of this suspension was mixed with 1 $\mu$g﻿ of expression construct DNA in chilled 2 mm electroporation cuvettes, and rapidly pulsed using a Bio-Rad GenePulser II (exponential pulse decay; 11 kV cm ${}^{-1}$ electric field strength; 50 $\mu$ F capacitance; 600 $\Omega$ resistance). After the pulse, cells were immediately transferred to 5 ml of media and recovered for 24 h at dim light and 25 ${}^{\circ }\mathrm{C}$. Cells were pelleted (2500×*g*, 10 min), resuspended in a small volume of supernatant and plated on ASW plates containing media and 5 $\mu$g﻿ ml ${}^{-1}$ zeocin. Colonies were screened after 4–5 weeks for high on-plate tdTomato fluorescence using a PathoScreen (PhenoVation Life Sciences, The Netherlands), via the RFP channel, transferred to liquid media containing zeocin and cultivated for 2 weeks to ensure complete removal of cells without resistance to zeocin, before streaking cultures on fresh agar plates.

### Flow cytometry analysis

Reporter fluorescence of tdTomato was quantified by flow cytometry analysis using an SH800S (Sony Biotechnology, USA) cell sorter, equipped with 488 nm and 561 nm lasers and a 70 or 100 $\mu$m nozzle microfluidic chip. Detector wavelengths for different channels were 488 nm for forward (gain 2) and side (gain 22%) scatter; $585\hspace{0.17em}\pm \hspace{0.17em}15$ nm for tdTomato (gain 45%) and $720\hspace{0.17em}\pm \hspace{0.17em}30$ nm for chlorophyll a (gain 40%). A minimum of 30,000 events were screened per sample. Gating was applied as previously described [59] to remove background noise. Only events in the gate “living cells” were considered for statistical evaluation.

### Western blot

Soluble protein was extracted from *N. oceanica* cultures during exponential growth phase. Approximately 3E9 cells were harvested (2500 g$\times$, 5 min), resuspended in 400 ﻿$\mu$l 0.075 mM Tris buffer (pH 8) and the microalgal suspension was bead beat for 3 cycles of 20 s at 2500 rpm, with 120 s pauses between cycles in a Lysing Matrix E (#116914500, MP Biomedicals) with a Precellys 24 homogenizer (Bertin Technologies). Then, tubes were frozen at -20 ${}^{\circ }\mathrm{C}$ for 90 min, thawed at 20 ${}^{\circ }\mathrm{C}$, frozen and thawed again, and pelleted (15,000*g*$\times$, 5 min). The supernatant was transferred to a fresh tube, and protein content was quantified by modified Lowry assay (DC Protein Assay, Biorad #5000116) with a BSA calibration standard. 45 $\mu$g of soluble protein was mixed with 5 $\times$ Laemmli reagent, boiled at 95 ${}^{\circ }\mathrm{C}$ for 5 min and separated by SDS-PAGE on 8–16% TGX protein gels (Biorad) with TGS running buffer for 40–50 min at 200 V. Subsequently, proteins were blotted onto PVDF membranes (Thermo Fisher Scientific #PB5310) using a Power Blotter XL system (Thermo Fisher Scientific #PB0013). Membranes were blocked with TBS-T containing 1% (w/v) skim milk powder (Biorad #170–6404), and incubated with anti HA antibody (500 $\times$ diluted, Thermo Fisher Scientific #26183) on a rocking shaker for 2 h at 22 ${}^{\circ }\mathrm{C}$, and then overnight at 4 ${}^{\circ }\mathrm{C}$. Then, membranes were washed thrice with TBS-T, incubated with an HRP-conjugated secondary antibody (2000 $\times$ diluted, Thermo Fisher Scientific #A10551) for 2 h, and washed thrice again. Chemiluminescence detection was done using an iBright CL1500 Imaging system (Thermo Fisher Scientific #A44114), with clarity Western ECL substrate (Biorad #170–5061) for 1 and 15 min. After detection, gels and membranes were Coomassie-stained using Coomassie G-250 (Biorad #161–0787) and Coomassie R-250 (Biorad #161–0436), respectively, and destained with H ${}_{2}$ O or several changes of a destaining solution (7% v/v acetic acid, 50% v/v methanol) followed by H ${}_{2}$ O, respectively, to confirm appropriate equal blotting efficiencies across samples.

### Physiological and biochemical characterization of transformants

For biochemical characterization, microalgae were grown as follows. Individual colonies were picked from agar plates, and cultivated for 2 weeks in liquid media at 150 $\mu \mathrm{mol}\hspace{0.25em}{\mathrm{m}}^{-2}\hspace{0.25em}{\mathrm{s}}^{-1}$ light intensity. Subsequently, cultures were set to an OD ${}_{750}$ of 0.1 and incubated in an Algem HT24 photobioreactor (Algenuity, UK) which was placed inside an HT Multitron Pro shaker, using 0.2% CO ${}_{2}$-enriched air and light intensity of 600 $\mu \mathrm{mol}\hspace{0.25em}{\mathrm{m}}^{-2}\hspace{0.25em}{\mathrm{s}}^{-1}$. After 3 days, cultures were diluted to OD ${}_{750}$ of 0.1 and grown for an additional 3 days at the same conditions, before being used to inoculate experimental cultures (data shown in Figs. 3–5). Experimental cultures were set to a starting OD ${}_{750}$ of 0.085, and incubated using the same cultivation conditions as for previous cultures. Growth curves were obtained by daily measuring of OD ${}_{750}$ and cell count, 1.5 h before onset of the dark phase of the 16:8 diurnal cycle. A linear model was fitted to logarithmically-transformed OD ${}_{750}$ values to obtain the maximum specific growth rate ${\mu }_{max}$ as the slope of the regression line. Photosynthetic performance was measured daily as described above, and averaged over the entire exponential growth phase for each flask. Cellular biovolume was measured using a Beckman Coulter Multisizer 3 (Beckman Coulter Inc., USA, with 50 ﻿$\mu$m orifice). Data shown in Fig. 3d are for the final day of the cultivation. After 3 days, microalgal cultures were harvested by centrifugation (4,000 × g, 10 min) and resuspended in 2 ml of ASW. 650 $\mu$l﻿ of the suspension was subjected to lipid quantification as described below. DCW of the suspension was measured as described before [60], using 0.5 M ammonium formate for washing. For N-starvation experiments, cultures were grown as described above, but harvested after 3 days of cultivation at 600 $\mu \mathrm{mol}\hspace{0.25em}{\mathrm{m}}^{-2}\hspace{0.25em}{\mathrm{s}}^{-1}$ by centrifugation ($2500\times$ g, 5 min), washed with ASW and resuspended in ASW supplemented with regular concentrations of all nutribloom ingredients, except for NO ${}_{3}$. Cultures were set to an OD ${}_{750}$ of 0.4, and cultivated for 2 additional days at 600 $\mu \mathrm{mol}\hspace{0.25em}{\mathrm{m}}^{-2}\hspace{0.25em}{\mathrm{s}}^{-1}$, before harvesting and processing as described above.

### Assessment of photosynthetic performance

The maximum efficiency of photosystem II photochemistry was quantified on 25 min dark-adapted samples using in vivo chlorophyll fluorescence analysis with an AquaPen AP 100-C (Photon System Instruments, Czech Republic) handheld fluorometer, according to the manufacturer’s protocol. Non-photochemical quenching, coefficient of photochemical quenching and development of quantum yield of photosystem II were measured using the NPQII protocol of AquaPen AP 100-C, with actinic and saturating light intensities of 1,000 and 3,000 $\mu \mathrm{mol}\hspace{0.25em}{\mathrm{m}}^{-2}\hspace{0.25em}{\mathrm{s}}^{-1}$, respectively. For NPQ/qP/QY measurements, microalgal cultures were analyzed after 2 days of incubation in N-replete media at 600 $\mu \mathrm{mol}\hspace{0.25em}{\mathrm{m}}^{-2}\hspace{0.25em}{\mathrm{s}}^{-1}$ illumination, and after 1 h of dark adaptation. Parameters were calculated by AquaPen AP 100-C software according to equations reported elsewhere [61].

### Quantification of non-polar and polar lipids via GC-FID

Non-polar and polar lipid content and FA profiles were determined using a modified version of the protocol described by Remmers and colleagues [62]. 650 $\mu$l﻿ of a microalgae suspension with known DCW concentration was subjected to FA extraction, separation into non-polar and polar lipids, methylation and quantification. After freeze-drying in a lysing matrix (#116,914,050-CF, MP Biomedicals), the biomass was subjected to mechanical cell disruption and lipid extraction using chloroform:methanol (1:1.25) containing the 2 internal standards tripentadecanoin (T4257; Sigma-Aldrich) and 1,2-didecanoyl-*sn*-glycero-3-phospho-(1*’*-*rac*-glycerol) (840,434, Avanti Polar Lipids Inc.) at known concentrations. Polar and apolar lipids were separated with Sep-Pak Vac silica cartridges (6 cc, 1000 mg; Waters). NLs were eluted with hexane:diethylether (7:1 v/v), and PLs with methanol:acetone:hexane (2:2:1 v/v). Solvents were evaporated under N ${}_{2}$ gas and lipids were methylated in 5% (v/v) H ${}_{2}$ SO ${}_{4}$-containing MeOH (1 h, 100 ${}^{\circ }\mathrm{C}$), extracted with hexane, and analyzed by gas chromatography using a Flame Ionization Detector (GC-FID, Agilent Technologies 7890B) equipped with a Nukol™ fused silica capillary column (Supelco, 30 m × 0.53 mm × 1.0 µm film thickness). Identification and quantification of FAs was done based on the relative responses of individual FAs compared to the peak area of internal standards (Merck #840434P and #T4257) and ultimately normalized to the DCW of the sample. Retention times and response factors of individual FAs were calibrated using external FAME standards (Merck #CRM18920 and #L6503-1G).

### Bioinformatical protein analysis

The *N. oceanica GPAT* gene model annotations of *NO03G04130* and *NO12G00610* and their amino acid sequences were retrieved from the current reference genome for *N. oceanica* IMET1v2 [63]. The two encoded proteins and the AoGPAT protein were analyzed for homologues in other organisms and for conserved domains using protein–protein BLAST on *Non-redundant protein sequences* with standard algorithm parameters, and by querying the conserved domain database of the NCBI [64]. Protein secondary structure and related features, as well as subcellular localization were predicted using the in silico prediction tool PredictProtein [65]. Transmembrane helices in NO03G04130 were analyzed by PredictProtein using the TMSEG algorithm [66], and further by MEMSAT-SVM [67], CCTOP [68], SPLIT4 [69] and TMPRED [70]. Prediction of the disordered domain in NO03G04130 by PredictProtein was confirmed by DISOPRED3 [71] and IUPred2A [72].

### Statistical data treatment

R statistical software [73] was employed for all data processing and statistical evaluation. Two-way ANOVA was used to test for significant main effects. In case of a significant ANOVA, means of multiple groups were compared using Tukey’s HSD test. Differences were considered significant in case of $p$<0.05. For quantitative flow cytometry analyses, the median of the fluorescence distribution of (at least 30,000) gated events in the FL2-A channel was taken as representative value for a single sample.

### Oligonucleotides and gene fragments

Oligonucleotides used in this study are shown in Additional file 5: Table S2.

## Supplementary Information

**Additional file 1: Fig. S1.** Schematic of the integration process for expression constructs and genotyping of transformant strains. (a) The expression constructs were targeted to the ribosomal DNA cistron on chromosome 3, which is a genomic safe harbor for strong Pol I expression. Endogenous 25S rDNA sequences were added between P_PolI and the IRES (I), and between T_PolI and the expression enhancer (T_(α-tub)), respectively. We found that this addition improved the efficiency of homologous recombination in previous experiments. The 25S rDNA sequences were omitted in Fig. 1 for simplicity. During homologous recombination, the expression constructs could insert by either terminal or internal crossovers between the cassette and the chromosome, resulting in two different configurations. We have previously shown that both configurations are equally suitable to drive strong expression of transgenes [34]. Successful insertion at the safe harbor locus was confirmed by PCR (b) using primers depicted as solid horizontal arrows. The elements are not drawn to scale to aid visualization. The expected length for amplicons is indicated for NoGPAT (AoGPAT). (b) Genotyping PCR confirmed construct insertion at the safe harbor site in all transgenic strains. In NoGPAT-M1, the construct was inserted by internal crossovers (ic), and in all other strains by terminal crossovers (tc). The control reaction (D) PCR product for NoGPAT-M2 was shorter than expected, due to partial deletion of the tdTomato coding sequence (revealed by sequencing).

**Additional file 2: Fig. S2.** Growth curves of N. oceanica NoGPAT and AoGPAT mutants during nitrogen deprivation. (a) Growth and final biomass densities of mutant strains was comparable to each other and to the wild type. (b) Maximum quantum efficiency of photosystem II photochemistry of transformants after ~2 days of exposure to nitrogen deprivation.

**Additional file 3: Fig. S3.** Fatty acid profiles of NLs and PLs for N. oceanica AoGPAT expression mutants. (a) FA compositions for exponentially growing cultures. No relevant differences are visible for any of the mutants compared to the wild type. (b) FA composition for cultures after exposure to 2 days of N-depletion. (a-b) Statistical significance was assessed by Tukey’s HSD test. (*): p<0.01.

**Additional file 4: Fig. S4.** Total PUFA contents of GPAT transformants during exponential growth phase, normalized to the DCW. (a) C18:2 was substantially enriched in biomass of NoGPAT and AoGPAT mutants M1 and M2. (a) Among all FA species, C18:3 showed the highest relative increase per DCW in NoGPAT M1 and M2. (c) C20:4 was enriched in NoGPAT-M1 and M2, but not in AoGPAT mutants. (d) C20:5 was elevated in NoGPAT-M1 and M2, albeit to a lesser extent than other PUFA species. (e) NoGPAT-M1 and M2 biomass was significantly enriched with PUFAs, which accounted for 6.8% of DCW. (a-e) Relative differences compared to the WT are indicated above groups. Statistical significance (N=3, N=5 for WT) was assessed by Tukey’s HSD test, in case of a significant ANOVA outcome. *p<0.05; **p<0.01; ***p<0.001.

**Additional file 5: Table S1.** Maximum specific growth rates, non-polar lipid (NL), total lipid (TL) and PUFA contents (meanSD), and productivities of NoGPAT and AoGPAT transformants. Relative changes compared to the wild type (WT) and statistical significance levels are given (highlighted in italics). Statistical significance was assessed by Tukey’s HSD test. *p<0.05; **p<0.01; ***p<0.001. **Table S2.** Oligonucleotides used in this study.

## Data Availability

All data produced in this study are presented in the article or additional material.
