# Supplementary figures and images for: Expression of glycerol-3-phosphate acyltransferase increases non-polar lipid accumulation in Nannochloropsis oceanica
Source: Microb Cell Fact. 2023 Jan 16;22:12. doi: 10.1186/s12934-022-01987-y (PMC9844033; doi:10.1186/s12934-022-01987-y)

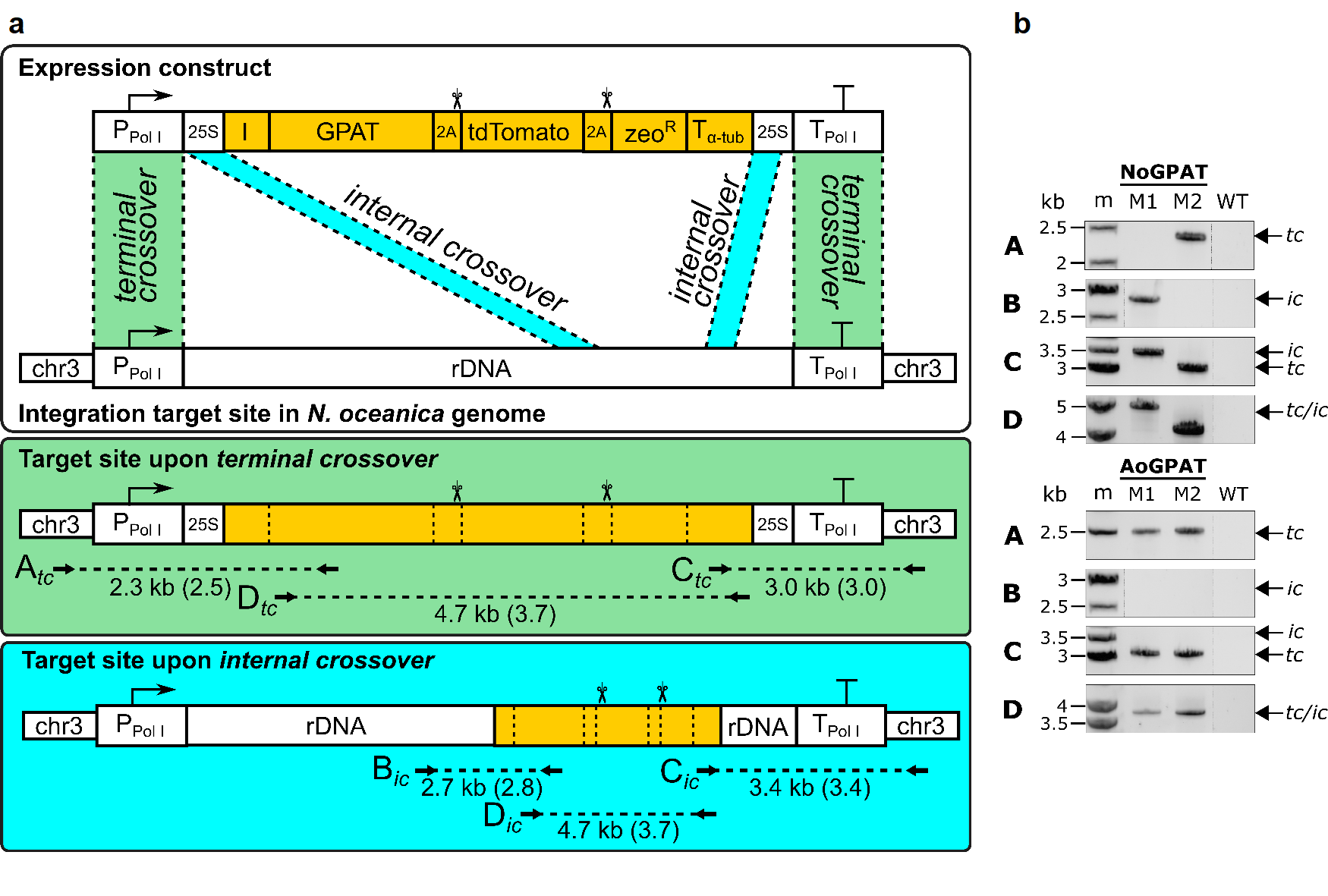

Supplement: Supplementary file 1 — Additional file 1: Fig. S1. Schematic of the integration process for expression constructs and genotyping of transformant strains. (a) The expression constructs were targeted to the ribosomal DNA cistron on chromosome 3, which is a genomic safe harbor for strong Pol I expression. Endogenous 25S rDNA sequences were added between P_PolI and the IRES (I), and between T_PolI and the expression enhancer (T_(α-tub)), respectively. We found that this addition improved the efficiency of homologous recombination in previous experiments. The 25S rDNA sequences were omitted in Fig. 1 for simplicity. During homologous recombination, the expression constructs could insert by either terminal or internal crossovers between the cassette and the chromosome, resulting in two different configurations. We have previously shown that both configurations are equally suitable to drive strong expression of transgenes [34]. Successful insertion at the safe harbor locus was confirmed by PCR (b) using primers depicted as solid horizontal arrows. The elements are not drawn to scale to aid visualization. The expected length for amplicons is indicated for NoGPAT (AoGPAT). (b) Genotyping PCR confirmed construct insertion at the safe harbor site in all transgenic strains. In NoGPAT-M1, the construct was inserted by internal crossovers (ic), and in all other strains by terminal crossovers (tc). The control reaction (D) PCR product for NoGPAT-M2 was shorter than expected, due to partial deletion of the tdTomato coding sequence (revealed by sequencing). [file 12934_2022_1987_MOESM1_ESM.tiff]

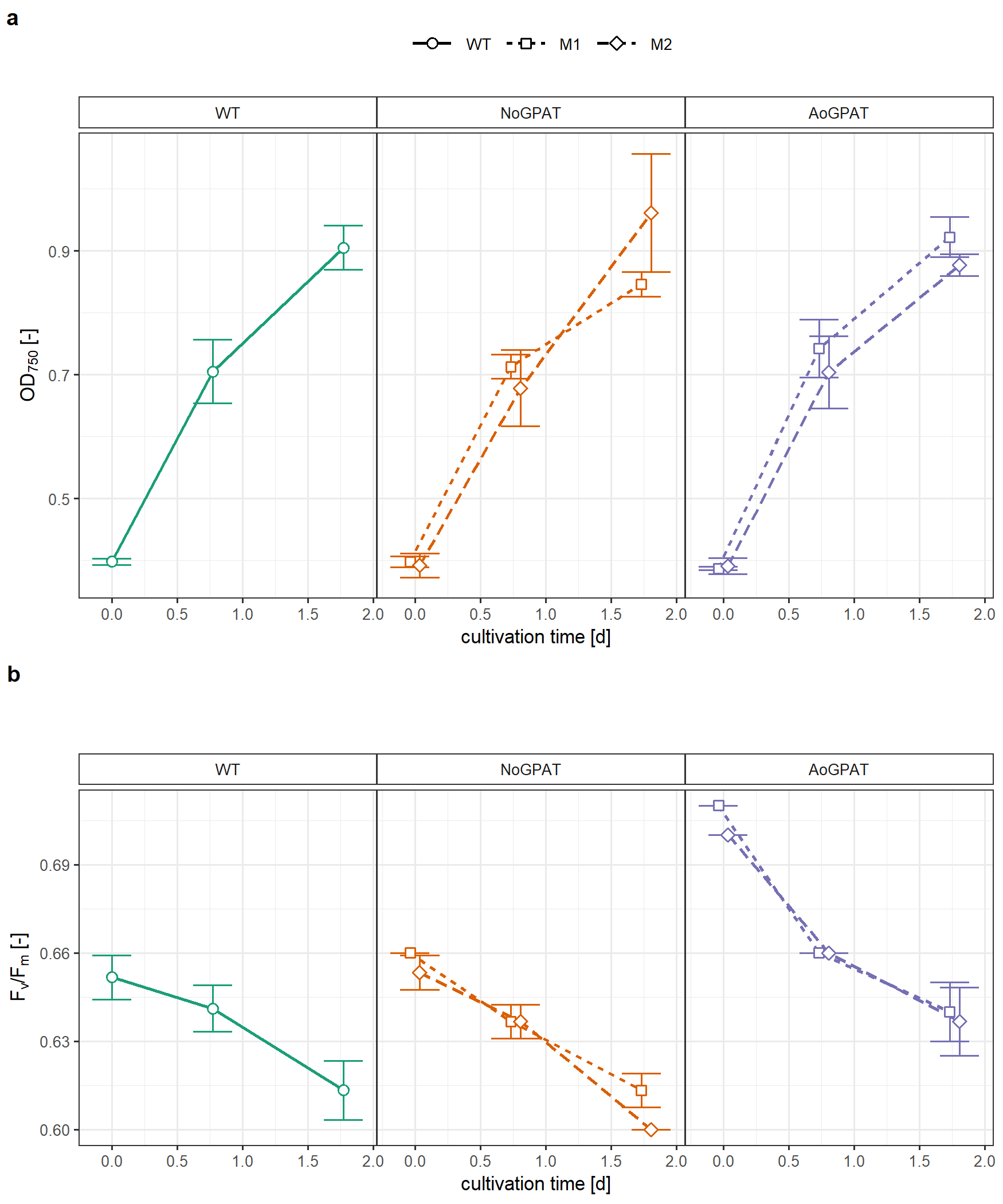

Supplement: Supplementary file 2 — Additional file 2: Fig. S2. Growth curves of N. oceanica NoGPAT and AoGPAT mutants during nitrogen deprivation. (a) Growth and final biomass densities of mutant strains was comparable to each other and to the wild type. (b) Maximum quantum efficiency of photosystem II photochemistry of transformants after ~2 days of exposure to nitrogen deprivation. [file 12934_2022_1987_MOESM2_ESM.tiff]

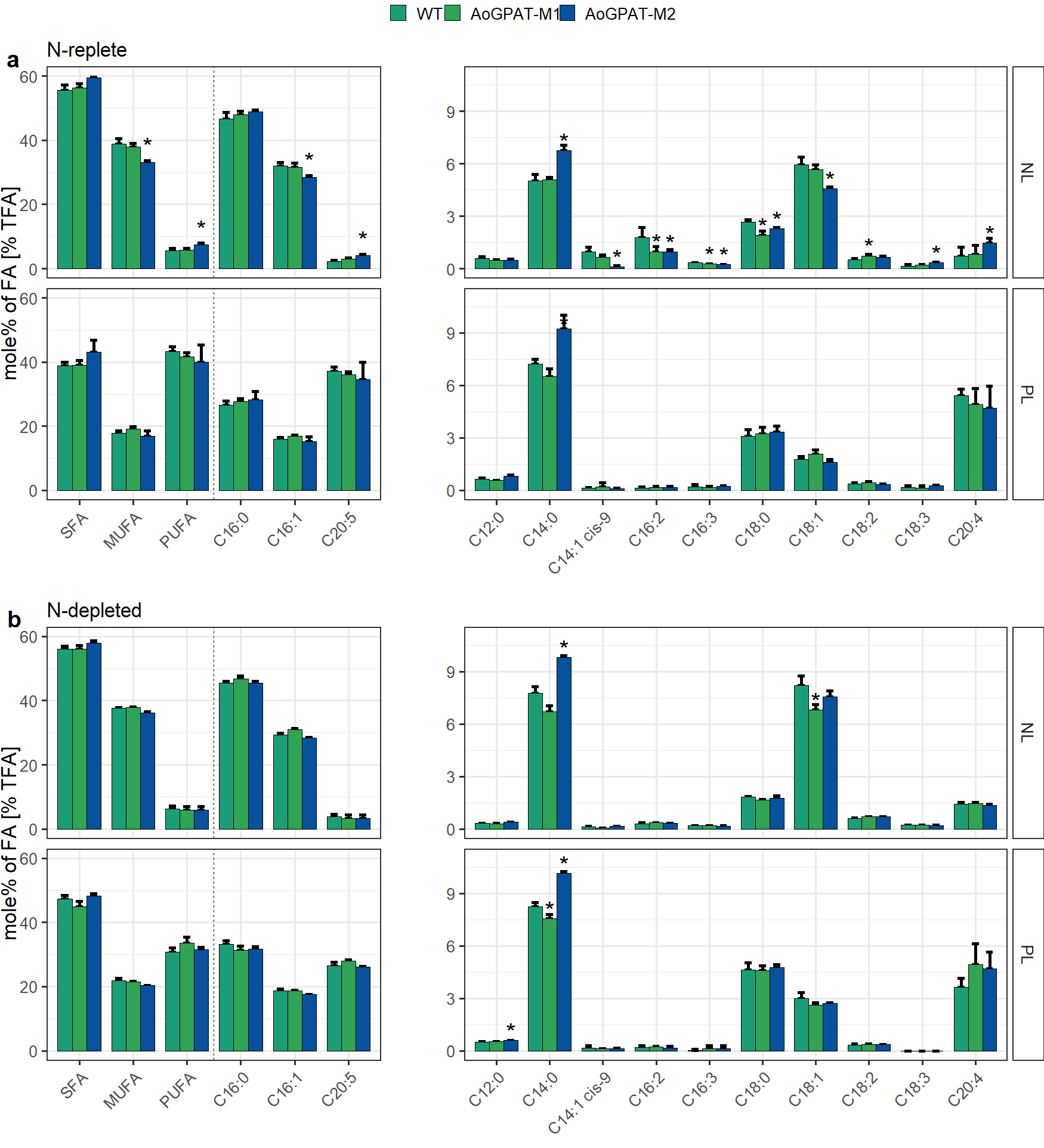

Supplement: Supplementary file 3 — Additional file 3: Fig. S3. Fatty acid profiles of NLs and PLs for N. oceanica AoGPAT expression mutants. (a) FA compositions for exponentially growing cultures. No relevant differences are visible for any of the mutants compared to the wild type. (b) FA composition for cultures after exposure to 2 days of N-depletion. (a-b) Statistical significance was assessed by Tukey’s HSD test. (*): p<0.01. [file 12934_2022_1987_MOESM3_ESM.tiff]

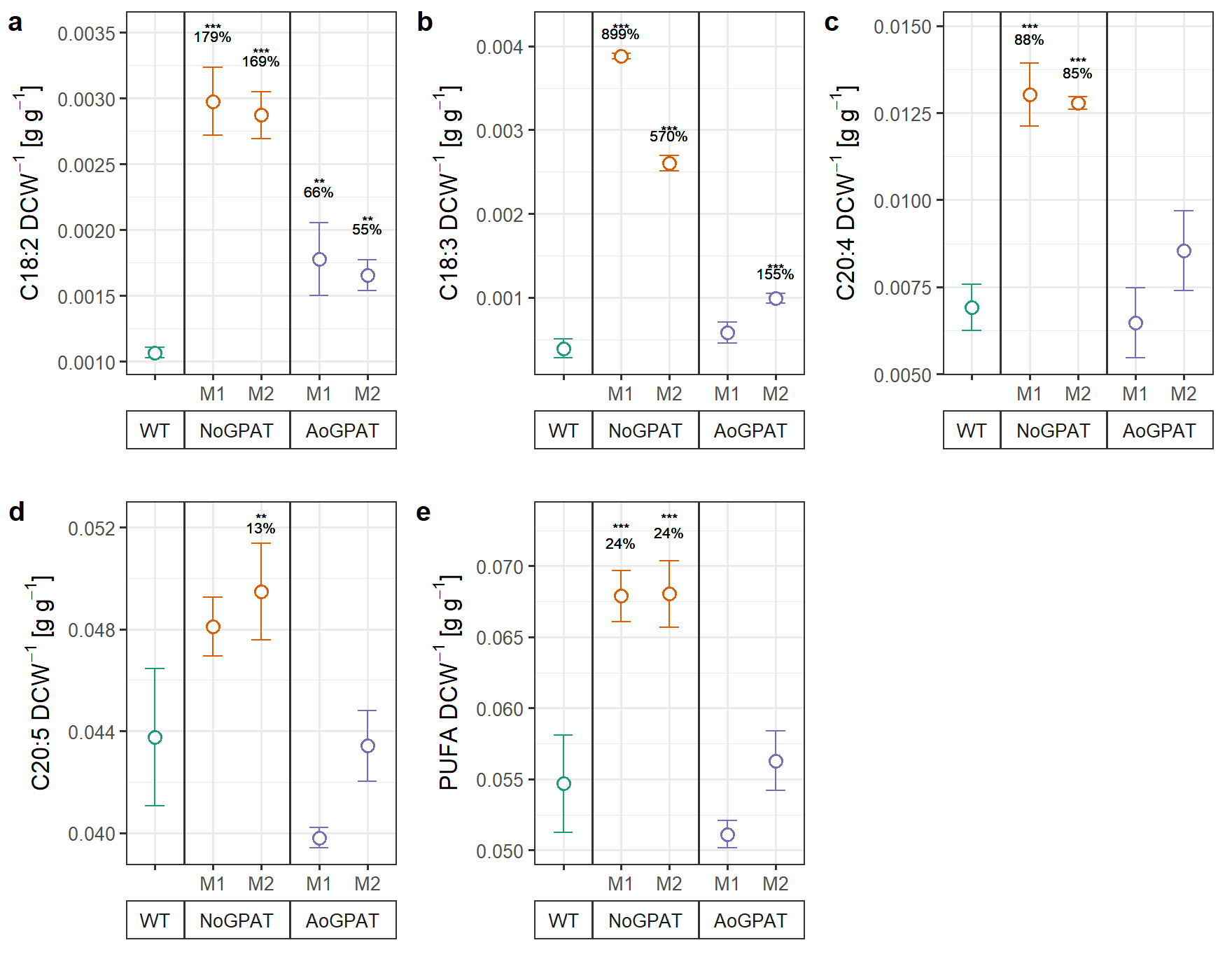

Supplement: Supplementary file 4 — Additional file 4: Fig. S4. Total PUFA contents of GPAT transformants during exponential growth phase, normalized to the DCW. (a) C18:2 was substantially enriched in biomass of NoGPAT and AoGPAT mutants M1 and M2. (a) Among all FA species, C18:3 showed the highest relative increase per DCW in NoGPAT M1 and M2. (c) C20:4 was enriched in NoGPAT-M1 and M2, but not in AoGPAT mutants. (d) C20:5 was elevated in NoGPAT-M1 and M2, albeit to a lesser extent than other PUFA species. (e) NoGPAT-M1 and M2 biomass was significantly enriched with PUFAs, which accounted for 6.8% of DCW. (a-e) Relative differences compared to the WT are indicated above groups. Statistical significance (N=3, N=5 for WT) was assessed by Tukey’s HSD test, in case of a significant ANOVA outcome. *p<0.05; **p<0.01; ***p<0.001. [file 12934_2022_1987_MOESM4_ESM.tiff]
