# Supplementary material for: Expression of glycerol-3-phosphate acyltransferase increases non-polar lipid accumulation in Nannochloropsis oceanica
Source: Microb Cell Fact. 2023 Jan 16;22:12. doi: 10.1186/s12934-022-01987-y (PMC9844033; doi:10.1186/s12934-022-01987-y)
Supplement: Supplementary file 5 — Additional file 5: Table S1. Maximum specific growth rates, non-polar lipid (NL), total lipid (TL) and PUFA contents (meanSD), and productivities of NoGPAT and AoGPAT transformants. Relative changes compared to the wild type (WT) and statistical significance levels are given (highlighted in italics). Statistical significance was assessed by Tukey’s HSD test. *p<0.05; **p<0.01; ***p<0.001. Table S2. Oligonucleotides used in this study. [file 12934_2022_1987_MOESM5_ESM.docx]

Table S1: Maximum specific growth rates, non-polar lipid (NL), total lipid (TL) and PUFA contents (mean$\pm$SD), and productivities of NoGPAT and AoGPAT transformants. Relative changes compared to the wild type (WT) and statistical significance levels are given (highlighted in italics). Statistical significance was assessed by Tukey’s HSD test. (*): $p<0.05$; (**): $p<0.01$; (***): $p<0.001$

| **Strain** | **µ_max_** | **Δ_µ-max_** | **NL**  **DCW^-1^** | **Δ_NL_** | **Δ_NL-prod._** | **TL**  **DCW^-1^** | **Δ_TL_** | **Δ_TL-prod._** | **PUFA**  **DCW^-1^** | **Δ_PUFA_** | **Δ_PUFA-prod._** |
| --- | --- | --- | --- | --- | --- | --- | --- | --- | --- | --- | --- |
| **WT** | 0.89±0.05 | 0% | 12.4±1.9 | 0% | 0% | 23.4±1.7 | 0% | 0% | 5.5±0.3 | 0% | 0% |
| **NoGPAT-M1** | 0.80±0.02 | -10% | 17.6±1.1 | 42% (**) | 26% | 29.4±1.1 | 26% (***) | 12% | 6.8±0.2 | 24% (***) | 11% |
| **NoGPAT-M2** | 0.90±0.06 | 1% | 16.9±0.9 | 36% (**) | 37% (*) | 28.6±0.8 | 22% (***) | 23% (*) | 6.8±0.2 | 24% (***) | 26% (*) |
| **AoGPAT-M1** | 0.79±0.03 | -11% | 18.7±0.2 | 51% (***) | 34% (*) | 28.0±0.5 | 20% (**) | 7% | 5.1±0.1 | -7% | -16% |
| **AoGPAT-M2** | 0.85±0.04 | -4% | 18.5±1.1 | 49% (***) | 42% (**) | 28.3±0.6 | 21% (***) | 16% | 5.6±0.2 | 3% | -1% |

Table S2: Oligonucleotides used in this study.

| ID | Sequence | Application |
| --- | --- | --- |
| oCSG001 | ATGGTGAGCAAGGGCGAGGA | Linearisation of pCS-EC6 as backbone for pCS-EC6-NoGPAT and pCS-EC6-AoGPAT (assembly fragment 1) |
| oCSG002 | CTGCTCGGAGGGGAGGATG | Linearisation of pCS-EC6 as backbone for pCS-EC6-NoGPAT and pCS-EC6-AoGPAT (assembly fragment 1) |
| oCSG003 | TCCCTTCATCCTCCCCTCCGAGCAGATGCCGTCTCGCAGCACCATC | Amplification of NoGPAT exon 1 (NoGPAT assembly fragment 2) |
| oCSG004 | GACTTTGACGGGGTTGGGATCAATGGCCAAAAGAATGAC | Amplification of NoGPAT exon 1 (NoGPAT assembly fragment 2) |
| oCSG005 | CATTGATCCCAACCCCGTCAAAGTCATCGCCGCCTCT | Amplification of NoGPAT exon 2 and HA tag (NoGPAT assembly fragment 3) |
| oCSG006 | GCGTAATCAGGCACATCGTAGGGGTAGGCGTCCTTCTTCCCCGTCGCCA | Amplification of NoGPAT exon 2 and HA tag (NoGPAT assembly fragment 3) |
| oCSG007 | TACCCCTACGATGTGCCTGATTACGCTATGACGACCCTGTCCTTCCA | Amplification of 2A encoding sequence (assembly fragment 4, both vectors) |
| oCSG008 | TGACCTCCTCGCCCTTGCTCACCATGGGCCCCGGGTTCTCCTC | Amplification of 2A encoding sequence (assembly fragment 4, both vectors) |
| oCSG009 | TCCCTTCATCCTCCCCTCCGAGCAGATGAATCGTTTGCTATTTGGTGTCTTC | Amplification of SP${}_{\mathrm{PDI}}$ (AoGPAT assembly fragment 2) |
| oCSG010 | AGGGGAGCGCCATGCTCCCATCCTTCTCAACGG | Amplification of SP${}_{\mathrm{PDI}}$ (AoGPAT assembly fragment 2) |
| oCSG011 | AAGGATGGGAGCATGGCGCTCCCCTGTCAG | Amplification of AoGPAT, ER retention signal and HA tag (AoGPAT assembly fragment 3) |
| oCSG012 | CGTAATCAGGCACATCGTAGGGGTAGAGCTCGTCCTTGTTGCGCATCAGCTCCACG | Amplification of AoGPAT, ER retention signal and HA tag (AoGPAT assembly fragment 3) |
| oCSG013 | GTTCGGAAACTATCGATAGGGTTTT | Amplification of linear expression constructs from pCS-EC6-NoGPAT and pCS-EC6-AoGPAT |
| oCSG014 | GAGTCCAGGGCACCCGAAAT | Amplification of linear expression constructs from pCS-EC6-NoGPAT and pCS-EC6-AoGPAT |
| oCSG015 | TCCCTTCATCCTCCCCTCCGAGCAGATGCCGTCTCGCAGCACCATC | Genotyping PCR NoGPAT mutants, control reaction (rxn D) |
| oCSG016 | GTTGGAAAGGGAGGGTAGTGGCGAT | Genotyping PCR NoGPAT mutants, control reaction (rxn D) |
| oCSG017 | CCGCGGTGTTGCGATGGCGCTCCCCTGTCAG | Genotyping PCR AoGPAT mutants, control reaction (rxn D) |
| oCSG018 | GTTGGAAAGGGAGGGTAGTGG | Genotyping PCR AoGPAT mutants, control reaction (rxn D) |
| oCSG019 | CAAGGGAGGCTGCGTAACACAACG | Genotyping PCR NoGPAT and AoGPAT mutants, 5’ reaction (rxn A) |
| oCSG020 | GACTTTGACGGGGTTGGGATCAATGGCCAAAAGAATGAC | Genotyping PCR NoGPAT mutants, 5’ reaction (rxn A/B) |
| oCSG021 | TGAACTGCAGGTTTGCCA | Genotyping PCR AoGPAT mutants, 5’ reaction (rxn A) |
| oCSG022 | GCTGCTTCTTCCTTCCATCCGTGACTGTAT | Genotyping PCR NoGPAT and AoGPAT mutants, 3’ reaction (rxn C) |
| oCSG023 | CTGTGTTTGAAGAAATGAGGATGCG | Genotyping PCR NoGPAT and AoGPAT mutants, 3’ reaction (rxn C) |
| oCSG024 | GACGAGTCTTCTAATCAAGGCA | Genotyping PCR, reaction for NoGPAT-M1, 5’ reaction (rxn B) |
| oCSG025 | GACTTTGACGGGGTTGGGATCAATGGCCAAAAGAATGAC | Genotyping PCR, additional reaction for NoGPAT-M1, 5’ reaction (rxn B) |
